# Children’s Single-Leg Landing Movement Capability Analysis According to the Type of Sport Practiced

**DOI:** 10.3390/ijerph17176414

**Published:** 2020-09-03

**Authors:** Isaac Estevan, Gonzalo Monfort-Torres, Roman Farana, David Zahradnik, Daniel Jandacka, Xavier García-Massó

**Affiliations:** 1 Activitat Física i Promoció de la Salut (AFIPS) Research Group, Department of Teaching of Musical, Visual and Corporal Expression, University of Valencia, 46022 Valencia, Spain; Xavier.Garcia@uv.es; 2 Human Movement Analysis Research Group (HUMAG), Department of Teaching of Musical, Visual and Corporal Expression, University of Valencia, 46022 Valencia, Spain; gonzalomonfort@gmail.com; 3 Education Unit, Florida Universitaria, 46470 Catarroja, Spain; 4 Department of Human Movement Studies, Human Motion Diagnostic Centre, University of Ostrava, 70100 Ostrava, Czech; Roman.Farana@osu.cz (R.F.); David.Zahradnik@osu.cz (D.Z.); daniel.jandacka@osu.cz (D.J.)

**Keywords:** motor development, motor control, drop jump landing, childhood, statistical parametric mapping

## Abstract

(1) **Background**: Understanding children’s motor patterns in landing is important not only for sport performance but also to prevent lower limb injury. The purpose of this study was to analyze children’s lower limb joint angles and impact force during single-leg landings (SLL) in different types of jumping sports using statistical parametric mapping (SPM). (2) **Methods**: Thirty children (53.33% girls, *M* = 10.16 years-old, standard deviation (*SD*) = 1.52) divided into three groups (gymnastics, volleyball and control) participated in the study. The participants were asked to do SLLs with the dominant lower limb (barefoot) on a force plate from a height of 25 cm. The vertical ground reaction force (GRF) and lower limb joint angles were assessed. SPM{*F*} one-way analysis of variance (ANOVA) and SPM{*t*} unpaired *t*-tests were performed during the landing and stability phases. (3) **Results**: A significant main effect was found in the landing phase of jumping sport practice in GRF and joint angles. During the stability phase, this effect was exhibited in ankle and knee joint angles. (4) **Conclusions**: Evidence was obtained of the influence of practicing a specific sport in childhood. Child volleyball players performed SLL with lower impact force and higher knee flexion than child gymnasts. Training in specific jumping sports (i.e., volleyball and gymnastics) could affect the individual capacity to adapt SLL execution.

## 1. Introduction

Physical activity and exercise are important for both health and growth [1,2], and it is associated with motor skill development [2,3]. Physical activity, sport and/or exercise fosters the underlying mechanisms of children’s and adolescents’ motor competence (e.g., coordination patterns or stretch-shortening cycle) [2,4,5] and facilitates better regulation and reduction of impact forces during practice [6]. In this regard, the acquisition of new motor patterns as a result of physical activity and exercise not only depends on training but also on other factors such as age [6], level of performance [5], or the type of physical activity and sport practiced [7].

Landing in its different forms, such as single-leg landing (SLL) [5,6,8,9,10] is frequently used as a motor task during physical activity and is commonly used for studying motor pattern development in addition to dynamic postural stability and injury prevention. Landing, an integral part of sports [11], is a discrete task with a different beginning and end, without a continuous cyclic pattern [12]. It requires the rapid absorption of impact forces and involves a large amount of strain on the ligaments of the lower limb joints [11], however landing seems to be practiced only in specific jumping sports [12] and is one of the most frequently examined techniques in gymnastics [13,14,15,16,17]. Understanding the motor patterns of landing is important not only for performance but also to prevent lower limb injuries [18].

In the type of physical activity and sports that involve frequent jumping and landing, training improves motor competence in stability and jump-landings [10,19], as shown by comparing individual motor patterns according to the level of performance [5]. Studies analyzing injury prevention and risk factors during landing (e.g., in the anterior cruciate ligament (ACL)) found that expert dancers had a better shock absorption mechanism in landing than non-dancer recreational athletes with a minimum of three hours per week of physical exercise [19]. Also, experienced triple jumpers activated their muscles earlier and to a greater extent during the pre-activation phase than inexperienced individuals who were physically active [20].

Age is another factor that can affect motor pattern development [6], although most studies in the field involved adults. Inconsistent results were found when attempting to determine the biomechanical factors involved in minimizing young adults’ impact forces and lower limb joint loading during landing. In healthy college students who regularly played sports and exercise two to three times per week for a total of 2–3 h without following a professionally designed training scheme, increasing the knee flexion angle on initial foot contact with the ground seems to be associated with reduced impact forces and knee loading in landing [18], although this is not conclusive and the same authors stated that at contact with the ground large hip and knee flexion do not necessarily reduce impact forces; in this line, small increases in knee flexion did not affect the level of ground reaction forces (GRF) in landing [21]. Smaller limb joint motion instead of joint configuration on initial foot contact with the ground is important for reducing landing impact forces in a stop-jump task [18]. Even though adults and children seem to have similar lower limb joint flexion-extension on landing [6], adults show higher pre-activation and muscle activation levels than children, which is seen as better movement control [6] and a protection mechanism [22]. From a developmental viewpoint, more consistent motor patterns are acquired with age [12].

Although the studies on children and/or adolescents during landing are limited, the existing evidence shows that adolescents with experience in a single sport displayed larger lower limb joint angle variability than their counterparts with less experience [7]. Early specialization was associated with less stability and a higher risk of injury on landing than in adolescents who practiced diverse sports [7]. Children around 7 years old with a mature jumping pattern had less lower hip flexion and higher knee flexion than less mature children [23]. The ability to perform single-leg balance appears to mature around 8–11 years of age [24]. However, further evidence indicates that physically active individuals do not have better motor control during landing [22]. Even though physical activity and sports practice seem to be important for health, an excessive focus on individual motor patterns from specializing in a single sport may disrupt motor development and coordination processes, which are acquired or refined at an early age [7]. As a result, studying children’s lower limb kinematics and kinetics may provide an insight into motor pattern development according to the type of sport practiced in childhood [25].

The literature in the field is limited as regards signal processing and data analysis. The procedure followed usually involves a reduced amount of information on joint angle and vertical GRF signals [25,26]. This is because a signal is a pool of numbers ordered according to the moment when they are acquired and classical statistical analysis includes only a few dependent variables. Some features are thus extracted from the signals to collect the most important information. A novel type of statistical analysis known as statistical parametric mapping (SPM) has now been developed which allows researchers to compare pictures (voxel by voxel) and analyze changes in magnetic resonance images, computerized axial tomography images and others related to pathological diagnosis [27,28]. SPM has been expanded to one-dimensional signals such as joint angles or GRF [29]. It can also detect the time or period of time of a whole motor task with differences between signals [30].

Keeping in mind that: (1) the lower limb joints’ coordination largely determines the dynamic stability of the leg [31]; (2) effective landing needs appropriate sagittal-plane lower limb joint angular motion to modulate GRF [32]; and (3) as most studies refer to physically active adults, it is not clear whether single-sport practice involving frequent jumping and landing in childhood influences motor pattern development and injury prevention [22]. The purpose of this study was, therefore, to analyze children’s lower limb joint angles in the sagittal plane and vertical GRF during SLL in different jumping sports using SPM. It is hypothesized that during SLL children practicing jumping sports will exhibit lower vertical GRF and different lower limb joint flexion-extension than their counterparts in the control group.

## 2. Materials and Methods

### 2.1. Participants

A prior sample size calculation was carried out using the data published by Keiner et al. [5] and revealed that 27 individuals were needed to detect an effect size *f* = 0.675 setting alpha and beta in 0.05 and 0.2, respectively. Thus, 30 children (14 boys and 16 girls) divided into three groups (10 children per group: gymnastics, volleyball, and none) participated in this study (mean age, weight, and height were 10.16 years, standard deviation (*SD*) = 1.52; 33.79 kg, *SD* = 10.93; and 1.39 m, *SD* = 0.11, respectively). The participants were recruited with two main inclusion criteria: (1) to have been regular players (at least training twice per week) of any of the aforementioned sports for at least a year, or not to practice any structured sport; (2) no injuries in the last six months. Gymnasts and volleyball players were recruited because each sport is an example of a jumping sport where and not where rules and regulations specifically limit the code of points in jumping, respectively. Participants in the control group were primary school children not involved in any organized sport. Socio-demographic and anthropometric characteristics are provided in Table 1. Boys and girls were included in each group since at ages between 6 and 13 they have similar balance control when performing single-leg balance and drop jumps [24]. The data for the whole sample were used in a previous paper focused on the reliability of data analysis [27]. Before participating in the experiment the children’s parents gave their written informed consent. The protocol was approved by the University Ethics Committee and was carried out in accordance with the principles of the Declaration of Helsinki (H1446557620395).

### 2.2. Instruments

The participants were asked to stand on a customized platform just behind a force plate (Kistler 9286AA^®^, Winterthur, Switzerland) on which they had to land. Forty-six reflective calibration markers were placed bilaterally on the lateral and medial malleoli, the medial and lateral femoral condyles, the greater trochanter and on the shoe/foot over the first and fifth metatarsal heads. Tracking and calibration markers were securely positioned to define the pelvis (iliac crest and posterior superior iliac spine, anterior superior iliac spine), the thighs and shanks (4 lightweight rigid plates holding a quaternion of markers), the shoe/foot (a triad of markers on the heel over the calcaneus) and the trunk (the acromion, the sternum–xiphoid process, the 10th thoracic vertebra and the 7th cervical vertebra). Kinematic data (lower limb joint angles) were synchronized with force data during the landing and stabilization (both collected at 240 Hz) with an eight-camera motion capture system (Qualisys Oqus^®^, Göteborg, Sweden). The cameras were placed around the force platform and offset with each other at an angle of 45 degrees and captured complete images of the participants.

### 2.3. Procedure

The participants were asked to land on their dominant lower limb (barefoot) on a force plate from a height of 25 cm with their hands on their hips. This specific height was established according to pilot data and consulted studies [17,33,34,35,36]. Lower limb dominancy was established by asking the children to kick a ball as hard as possible twice and the leg used was considered to be dominant (27 were right-footed and 3 left-footed). After landing they had to maintain single-leg balance for at least 8 s.

The children were allowed to warm up individually, including: joint mobility, jogging, stretching and small jumps, in addition to a specific warm-up that consisted of two-four trials to familiarize themselves with the landing process (total warm-up time around 8–10 min). A research assistant guided by using story-telling and supervised the warm up that was conducted mainly in a sports pavilion next to the laboratory where measures were conducted. Throughout the test, each participant was asked to step from the height with the landing leg on the force plate from the platform. The free leg was kept in the rear with the knee flexed approximately 90°. The trials were video recorded to reject those which: (i) started jumping instead of stepping; (ii) the free knee was not flexed around 90; (iii) the free leg touched the floor during the subsequent 8 s [25].

**Figure 1:**
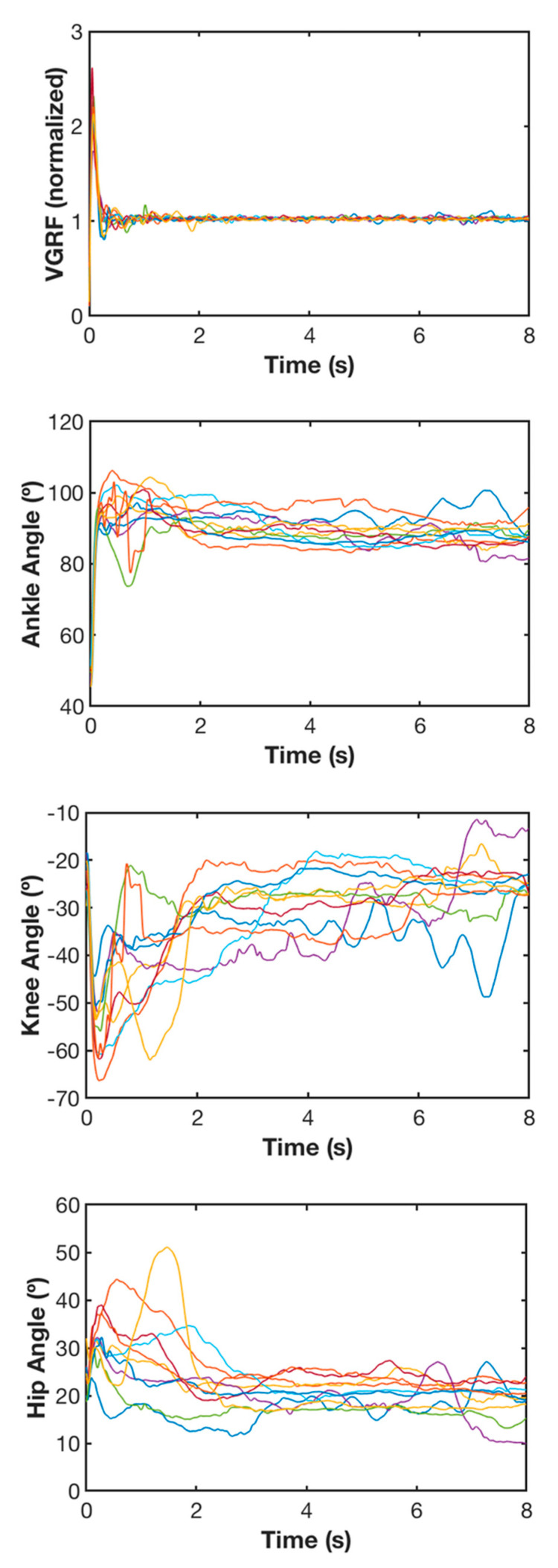
Example of a participant’s vertical ground reaction force (VGRF) normalized according to the body weight; and ankle, knee and hip angles in the sagittal plane expressed in degrees for the 10 trials. Each color line represents a trial.

Each trial started preferably between 1–3 s after the research assistant gave the appropriate signal to land on their dominant leg and keep their balance while remaining as still as possible for 8 s after landing and a 1–2 min rest interval was allowed between trials. Each child performed 10 successful trials. An example including a participant’s trials is exhibited in Figure 1.

### 2.4. Data Analyses

Data from the markers were processed using Visual 3D software (C-motion^®^, Victory Blvd Burbank, CA, USA). Signals started based on the threshold value of 15 N for the magnitude of GRF. Marker histories and vertical GRF were smoothed with a fourth-order, 12 Hz low-pass Butterworth filter. All extremity segments were modelled as a frustum of right circular cones, and the torso and pelvis were modelled as cylinders. The angular displacement of the pelvis, thigh, calf and foot segments was measured in each of the three planes (sagittal, frontal and transverse) but only sagittal plane data were used in the current study; the positive and negative signs describe the direction of the vector. Vertical GRF was normalized by body weight.

### 2.5. Statistical Analyses

Matlab 2019a (Mathworks Inc., Natick, MA, USA) and the open-source spm1d package (v. M.0.4.1, [37]) were used to conduct the statistical analysis. To test the influence of jumping practice on the kinetics and kinematics of single-leg drop jump several Statistical Parametric Maps (SPM){*F*} one-way analyses of variance (ANOVA) were performed. When significant effects were found the follow-up was carried out by means of SPM{*t*} unpaired *t*-tests. In the SPM analysis, a conventional *F*- or *t*-statistic is calculated for each point of the landing curve. A critical threshold is computed using Random Field Theory and the condition that only the 5% of random smooth data is expected to cross it (i.e., the alpha level of significance is set at *p* = 0.05 controlling the type I error rate [38]).

Overall, a principal effect of the jumping sports practice (i.e., gymnastics, volleyball or none) at each time point of the curves is determined when the SPM{*F*} crosses the threshold. The pairwise comparisons (i.e., gymnastics vs. volleyball, volleyball vs. none, and gymnastics vs. none) revealed significant differences between groups when the SPM{*t*} crossed the threshold. The differences between the groups are thus determined at specific time points on the curves, which give a better overview than using summarizing features and traditional statistical analysis. To visualize the data, the analysis of the first second (landing period/impact phase) and the rest of the signals (stability period/phase) are reported independently. We chose one second because it is long enough to include the impact force and reach relative stability in the signals [25].

## 3. Results

### 3.1. Vertical Ground Reaction Force

**Figure 2:**
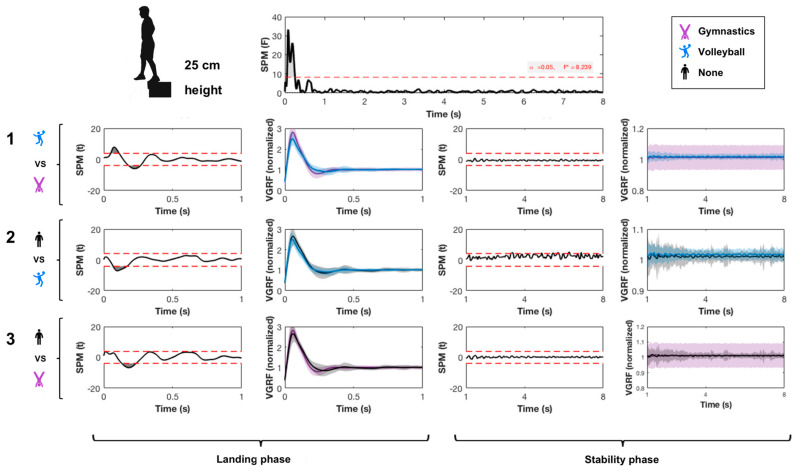
Effect of sports practice on vertical ground reaction force (GRF). SPM refers to statistical parametric mapping. Data acquired during landing and stability phases of single-leg landing from 25 cm height for 8 s are shown and compared. 1 refers to the pairwise comparison between gymnastics and volleyball groups; 2 refers to the pairwise comparison between volleyball and control groups; 3 refers to the pairwise comparison between gymnastics and control groups.

SPM{*F*} showed a significant main effect of jumping practice during SLL only during the impact phase (Figure 2). Pairwise comparisons showed that the impact and rebounds were higher in the gymnastics than in the volleyball and control groups. A lower impact force was found in volleyball than in the control group.

### 3.2. Lower Limb Joint Angles

**Figure 3:**
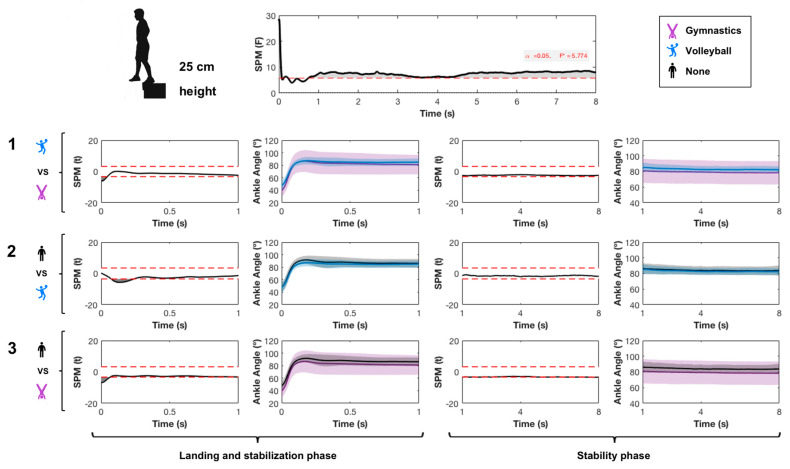
Effect of sports practice on ankle angle in the sagittal plane. SPM refers to statistical parametric mapping. Data acquired during landing and stability phases of single-leg landing from 25 cm height for 8 s are shown and compared. 1 refers to the pairwise comparison between gymnastics and volleyball groups; 2 refers to the pairwise comparison between volleyball and control groups; 3 refers to the pairwise comparison between gymnastics and control groups. An angle higher than 90° indicates plantarflexion while an angle lower than 90° indicates dorsiflexion.

**Figure 4:**
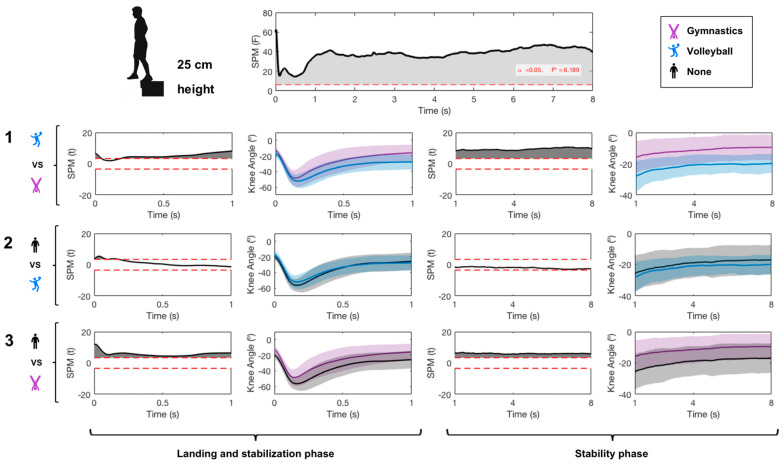
Effect of sports practice on knee angle in the sagittal plane. SPM refers to statistical parametric mapping. Data acquired during landing and stability phases of single-leg landing from 25 cm height for 8 s are shown and compared. 1 refers to the pairwise comparison between gymnastics and volleyball groups; 2 refers to the pairwise comparison between volleyball and control groups; 3 refers to the pairwise comparison between gymnastics and control groups. An angle lower than 0° indicates knee flexion.

**Figure 5:**
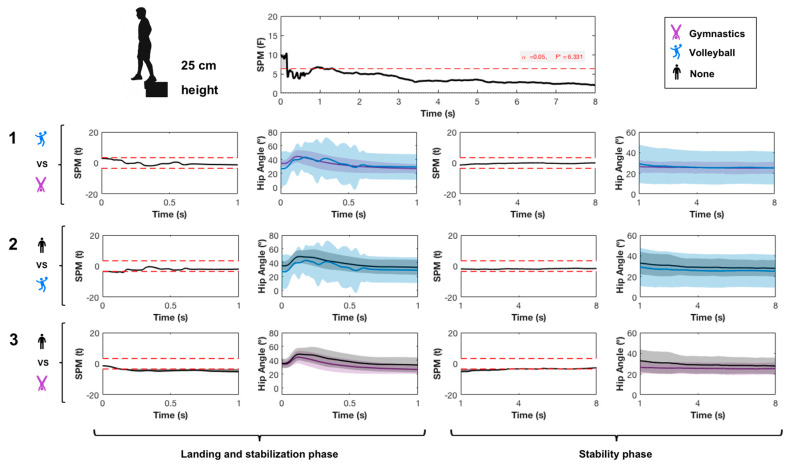
Effect of sport practice on hip angle in the sagittal plane. SPM refers to statistical parametric mapping. Data acquired during landing and stability phases of single-leg landing from 25 cm height for 8 s are shown and compared. 1 refers to the pairwise comparison between gymnastics and volleyball groups; 2 refers to the pairwise comparison between volleyball and control groups; 3 refers to the pairwise comparison between gymnastics and control groups. An angle higher than 0° indicates hip flexion while an angle lower than 0° indicates hip extension.

A significant main effect was found from jumping practice in the ankle (Figure 3), knee (Figure 4) and hip joint (Figure 5) and in the ankle joint angle during both the landing and stability phases. The gymnastics and volleyball groups had lower ankle dorsiflexion than the control group on landing. The gymnastics group had higher plantarflexion than the volleyball group before impact.

In the knee joint, the gymnastics group showed higher knee extension than the volleyball and control groups in both landing and stability phases. Pairwise comparisons showed a small difference between gymnastics and volleyball groups in the hip joint only during the landing phase. The gymnastics group had higher hip flexion than volleyball players.

## 4. Discussion

The analysis of SLL in children in different types of jumping sports can help in understanding the underlying mechanisms that drive children’s movement execution in terms of injury prevention and motor performance and the consequences for their health [4,5]. The purpose of this study was thus to analyze children’s lower limb joint angles in the sagittal plane and vertical GRF during SLL according to the type of jumping sport practiced (gymnastic, volleyball and a control group) using SPM. In the landing phase, while gymnasts performed SLL and achieved the highest vertical GRF, volleyball players obtained lower values than the control group. The gymnasts also used greater ankle plantarflexion and lower hip and knee flexion than the volleyball players. The main effect of the type of jumping sport practiced showed that the Gymnastic and Volleyball groups obtained more heterogeneous results than the control group.

Studies analyzing impact forces used by gymnasts have reported similar results to those found in the current study [17], in which prepubertal gymnasts had higher peak vertical GRF during landing than the untrained group, in agreement with Seegmiller and McCaw [14], who found that female gymnastics had higher peak vertical GRFs than female recreational athletes in drop landing. According to these results, high vertical GRFs experienced by gymnasts on landing may contribute to the risk of lower limb injuries. Volleyball players showed lower vertical GRF than the gymnastic and control groups, regardless of body weight. This supports previous findings which concluded that volleyball players seem to be able to absorb an impact force between 1.9 to 4.0 times their body weight [31,39,40]. These results might be related to lower limb movement, wherein a negative correlation between knee flexion and the magnitude of the peak GRF is found [41,42]. This could be also due to the nature of volleyball wherein, rather than gymnastics during landing, volleyball players must control their GRF quickly in order to react to any other unexpected stimuli incoming from the game [43]. Even though a study in volleyball suggested that impact force attenuation depended on the height of the drop [39], these results indicate that volleyball players have fewer lower limb injuries because the game seems to be associated with strategies of impact force attenuation.

The impact force on landing can be controlled by motor patterns such as stiff landing, which requires an erect body posture and is associated with high vertical GRF [42]. The higher ankle plantarflexion and knee extension in gymnastics than in volleyball players we found in our study could be seen as an example of ankle muscle stiffness for landing. In jumping sports, motor patterns involving lower limb joint kinematics also seem to be used for reducing vertical GRF on landing, which requires a high degree of hip flexion [16]. When gymnasts land with high hip flexion, they also have a tendency to lower vertical GRF and higher ankle and knee flexion [16]. Knee flexion has also been recognized as a key aspect for injury prevention during landing [44]. Studies on modulating the effects of different degrees of knee flexion on impact using controlled comparisons of ‘stiff’ and ‘soft’ landing techniques, confirmed the permitted maximum knee flexion on initial ground contact as a key landing aspect [44] and found an inverse relationship between the degree of initial or maximum knee flexion and the resulting peak GRF. Similar results were found in volleyball, when verbal instructions to increase knee flexion reduced the vertical GRF [45]. In our results, gymnasts showed lower hip flexion than volleyball players, and greater knee extension and lower ankle dorsiflexion than the volleyball and control group, taking into account that gymnasts’ landing strategy seems to be influenced by long-term and sport-specific training, which produces a stiffer landing pattern [17]; and that, in gymnastics, training background and specification make gymnasts capable of controlling high levels of angular momentum, which is associated with complex gymnastic skills, which could explain their more restricted joint flexion; instructors should therefore encourage young gymnasts to increase their lower limb joint flexion on landing since it might help to reduce high vertical GRF, align knee and pelvis stability [46] which could mean a decrease in the risk of falling with a consequent benefit in performance, and reduction of lower limb injuries [32].

The more diverse the motor tasks children experience the better their motor enrichment and repertoire [4,47]. The diverse experience of fundamental movement skills in the early years thus can lead to improved ability to perform complex movements later in life [4,48]. Early specialization can limit children’s motor development due to the impact of environmental restrictions (e.g., rules and regulations). This could be the case of gymnastics, wherein for instance, the greater ankle plantarflexion and lower knee joint flexion in gymnasts could be due to the restrictions in gymnastic landing techniques, with the desired minimum knee flexion [49]. Therefore, it seems that the gymnastic code of point influences training in terms of reducing knee, hip joint and ankle plantar flexion. Specific compensatory training to reduce vertical GRF [14,50,51] based on soft landing techniques [52] should be performed. This supports our previous recommendation to familiarize children with jumping sports wherein the code of point does not constrain or limit athletes’ technique such as volleyball. Maybe influenced by specific jumping training involving ball hitting, volleyball players seem to unconscious use a combination of the trunk lean in the sagittal plane with hip flexion on landing after a spike movement [53]. The combination of sport-specific and non-specific training based on trunk lean and hip flexion could, therefore, help children to reduce impact forces on landing.

As stated above, heterogeneous results are obtained when comparing control with gymnastic and volleyball groups. In vertical GRF, the untrained children in the control group obtained lower values than gymnasts and higher values than volleyball players. In terms of joint angles, the individuals in the control group showed higher ankle dorsiflexion than gymnasts and volleyball players, and lower knee flexion than gymnasts, but similar to volleyball players and a similar range of hip movement than their counterparts in the gymnastic and volleyball groups. These heterogeneous results could be seen as evidence of the children’s capacity to adapt their SLL to specific sports. While the control group’s vertical GRF was not as high as the gymnasts, they were not able to absorb as much GRF as the volleyball players. According to Newell [54], organized training with the appropriate information may lead to being able to adapt the performance to the established goal (e.g., injury prevention, soft landing, etc.). Children by themselves do not seem able to organize attempts to generate effective movement solutions, so coaches, practitioners or physical education teachers should encourage their motor development [55].

Regarding the practical implications of this work, even though gymnastics and volleyball are jumping sports, the consequences of landing can be divided into terms of injury prevention and performance. In the case of volleyball, almost half the landings are classified as SLL [56,57]. A study by Yeow, Lee, and Goh [58] showed that SLL increases landing-related injuries (i.e., ACL) compared to double-leg landing. SLL increased peak ACL force and reduced knee flexion and time to peak ACL strain, compared to double leg landings [59,60,61]. The incidence of ACL injuries in volleyball is greater among females than males [62]. The higher incidence of ACL could be influenced by the different kinetics and kinematics of landing skills. Females showed greater anterior shear force [63] and greater knee moments in the sagittal plane than males on landing [63,64] and landed with less knee flexion [64]. Even though the current training process in the juvenile volleyball categories concentrates on obtaining the maximal jump height and offensive skills, young volleyball players are not taught how to land after spike or block movement, which would give them greater knee flexion and reduce injuries (i.e., ACL).

Decker et al. [65] reported that it has generally been accepted that the internal and external loads experienced on landing in gymnastics may be controlled by the lower extremity kinematics. Taking into account that: (1) long-term gymnastics training induces modifications in children’s landing pattern and a stiffer lower limb strategy, as revealed by the shorter braking phase and smaller flexion joint angles [17]; (2) in gymnastics SLL is a specific task mainly performed during leaps and on a balance beam, especially in females; (3) it was observed that during training a preferred leg often develops when aiming to achieve skill consistency and reliability, which, from an injury prevention perspective can lead to a potential functional limb imbalance. Bradshaw [66] obtained inspiring results, with 11 out of 25 elite Australian gymnasts displaying functionally symmetrical drops and much lower overall levels of asymmetry. Asymmetry of the lower extremity in SLL is also associated with landing-related injuries [67] and is frequently seen in gymnasts of all levels on different types of apparatus including vault landings. Attenuating the force on one leg increases the risk of chronic overuse injury and is a common source of injury in other sports [68]. A combination of neuromuscular training, balance task progression and appropriate progress in foundational strengthening exercises including proper hip, knee and ankle positions (e.g., SLL progressions) should thus be the basis of gymnastic training. As developing local joint function, kinematic and kinetic chain integration in foundational SLL movement patterns may significantly increase neuromuscular control during specific gymnastic tasks, it is important to use SLL progression as part of children’s training or rehabilitation programs.

One of this study’s strengths is that it deals with a movement that had not previously been widely studied in children. As far as we know, there are no studies that analyze movement by the SPM method used in this work. We believe that these comparisons can be useful to determine how certain movements and sports influence the risk of injuries and muscle development. It should be noted that some of the differences we found between the groups did not happen exactly at the impact force. These differences had not been found in traditional analyses. One of the paper’s strengths is that SPM revealed differences in kinetic and kinematic parameters during the whole task and not only at certain time points. Nonetheless, this study is not devoid of limitations. Firstly, this is a cross-sectional study, so no causal inference can be assumed. Secondly, as the training hours per week were not recorded nor the physical activity participation, it is not possible to associate the findings of the study to the participants’ training load experience. In relation to the protocol, in order to control individual landing technique 10 successful trials were recorded. This means participants performed more than 10 trials but the actual number was not recorded. Having the exact number required to obtain the 10 successful trials would help us to know how difficult single-leg landing is for each group of participants. Another limitation is that we did not analyze movement coordination. Since hip and knee flexion are key aspects of injury prevention on landing [44], future studies should analyze coordination of the lower limb joints at different times to find the differences and determine their influence.

## 5. Conclusions

This study provides evidence in terms of GRF and lower limb joint flexion-extension of the influence of practicing a specific sport in childhood. Children who practice volleyball performed SLLs with lower impact forces and higher knee flexions than gymnasts. Unlike children who do not practice any structured sport, playing a specific jumping sport (e.g., volleyball and gymnastics) could influence the ability to adapt SLL performance. In addition, gymnastics or volleyball with specific environmental restrictions such as rules and regulations could diversify the experiences that enrich children’s SLL ability, not only for improving their performance but also to prevent injuries.

## Figures and Tables

**Table 1:** Participants’ characteristics according to the group (gymnastics, volleyball and control groups).

	Gymnastics		Volleyball		Control	
	Mean	*SD*	Mean	*SD*	Mean	*SD*
Age (years)	10.18	1.39	10.10	1.56	10.05	1.57
Weight (kg)	28.1	3.13	35.63	13.69	34.85	10.85
Height (m)	1.36	0.07	1.39	0.14	1.41	0.11
BMI (kg/m^2^)	15.20	1.15	17.82	3.34	18.02	3.34
Fat (%)	8.79	2.57	18.29	9.34	17.51	6.51
Sport time (Years)	4.3	1.73	2.8	1.86	-	-

Note. BMI refers to body mass index. *SD* refers to standard deviation.
